# Association between Cardiac Remodeling and Metabolic Alteration in an Experimental Model of Obesity Induced by Western Diet

**DOI:** 10.3390/nu10111675

**Published:** 2018-11-05

**Authors:** Artur Junio Togneri Ferron, Fabiane Valentini Francisqueti, Igor Otávio Minatel, Carol Cristina Vágula de Almeida Silva, Silméia Garcia Zanati Bazan, Koody André Hassemi Kitawara, Jéssica Leite Garcia, Camila Renata Corrêa, Fernando Moreto, Ana Lucia A. Ferreira

**Affiliations:** 1 São Paulo State University (Unesp), Medical School, Botucatu 18618-687, Brazil; fabianevf@gmail.com (F.V.F.); carolvagula@gmail.com (C.C.V.d.A.S.); sgzanati@fmb.unesp.br (S.G.Z.B.); kodiro@gmail.com (K.A.H.K.); jessleitegarcia@gmail.com (J.L.G.); correa.camila9@gmail.com (C.R.C.); fer_moreto@yahoo.com.br (F.M.); ferreira@fmb.unesp.br (A.L.A.F.); 2 São Paulo State University (Unesp), Institute of Biosciences, Botucatu 18618-689, Brazil; igorminatel@hotmail.com

**Keywords:** dyslipidemia, obesity, cardiac remodeling

## Abstract

The high consumption of fat and sugar contributes to the development of obesity and co-morbidities, such as dyslipidemia, hypertension, and cardiovascular disease. The aim of this study was to evaluate the association between dyslipidemia and cardiac dysfunction induced by western diet consumption. Wistar rats were randomly divided into two experimental groups and fed *ad libitum* for 20 weeks with a control diet (Control, *n* = 12) or a high-sugar and high-fat diet (HSF, *n* = 12). The HSF group also received water + sucrose (25%). Evaluations included feed and caloric intake; body weight; plasma glucose; insulin; uric acid; HOMA-IR; lipid profile: [total cholesterol (T-chol), high-density lipoprotein (HDL), non-HDL Chol, triglycerides (TG)]; systolic blood pressure, and Doppler echocardiographic. Compared to the control group, animals that consumed the HSF diet presented higher weight gain, caloric intake, feed efficiency, insulin, HOMA-IR, and glucose levels, and lipid profile impairment (higher TG, T-chol, non-HDL chol and lower HDL). HSF diet was also associated with atrial-ventricular structural impairment and systolic-diastolic dysfunction. Positive correlation was also found among the following parameters: insulin versus estimated LV mass (*r* = 0.90, *p* = 0.001); non-HDL versus deceleration time (*r* = 0.46, *p* = 0.02); TG versus deceleration time (*r* = 0.50, *p* = 0.01). In summary, our results suggest cardiac remodeling lead by western diet is associated with metabolic parameters.

## 1. Introduction

Obese individuals are typically predisposed to an increased heart rate and stroke volume, which may lead to ischemic cardiomyopathy, compensatory left ventricular remodeling, non-ischemic dilated cardiomyopathy, cardiac fibrosis, and apoptosis [1]. Furthermore, obesity is associated with metabolic syndrome (MS), a clustering of risk factors that includes visceral obesity, dyslipidemia, hyperglycemia, and hypertension. Among these factors, dyslipidemia is the major risk factor for cardiovascular disease (CVD). Although obesity influences blood lipid and lipoprotein levels, dietary factors are also able to influence these parameters [2].

Western diets (WD), which are rich in fat and carbohydrates may be responsible for the obesity epidemic, especially in industrialized countries [1]. WD are characterized by the consumption of high caloric-dense foods, and unbalanced proportions of fat (saturated vs unsaturated) and carbohydrates (high glycemic vs low glycemic), eating habits associated with an increased CVD risk [3]. Sonestedt et al. observed that individuals with low sucrose intake had lower triglycerides and higher HDL concentrations compared to those with high sucrose consumption [2]. Moreover, it has been already confirmed that the consumption of high carbohydrate foods and beverages increases the risk of MS [4]. 

There are several spontaneous (genetic) rodent models for MS that present cardiovascular disorders, including Zucker diabetic fatty rats [5,6], Goto-Kakizaki rats [7], and spontaneously hypertensive rats [8]. Although these models provide important information regarding the pathogenesis and the treatment of some aspects of MS, they do not reflect the diet-induced human metabolic syndrome [9]. Therefore, appropriate animal models mimicking human metabolic syndrome and related CVD are necessary to investigate the causes and progression of this disease and potential pharmacological interventions [10,11].

Experimental studies regarding cardiac function and diet-induced obesity present divergent results. Rats fed hypercaloric diets for 8 to 14 weeks developed obesity [12,13,14], but not cardiac disfunction, as assessed by echocardiogram [13,14]. However, Panchal et al. [15] showed ventricular dilation, increased systolic volume and increased estimated left ventricular mass in rats fed with high-carbohydrate and high-fat diet for both 8 and 16 weeks. Moreover, other researchers found diastolic dysfunction in isolated heart and papillary muscles [16], reduction in diastolic compliance [17] and impaired mechanical function of ventricular myocytes [18] from Ob rabbits [12,18] and rats [19] fed high-fat diets for 12 weeks. In this way, this study was designed to verify the hypothesis that our proposed diet model is able to induce cardiac remodeling. So, the aim of this study was to evaluate the effect of a Western diet on cardiac remodeling and its association with metabolic parameters.

## 2. Materials and Methods

### 2.1. Animals and Experimental Protocol

All the experimental protocol was approved by the Ethics Committee on the Use of Animals (CEUA) from Botucatu Medical School, São Paulo State University (UNESP), under number 1196/2016. Male Wistar rats (5–6 weeks old, weighing 209 ± 18g, *n* = 24) were obtained from the Animal Center of Botucatu Medical School, São Paulo State University, UNESP (Botucatu, SP, Brazil) and randomly divided into 2 experimental groups to receive chow diet (control group, *n* = 12) or high-sugar high-fat diet (HSF, *n* = 12) for 20 weeks. Animals were individually housed in temperature-controlled and 12-hour light-dark conditions. Diets were designed in our laboratory and previously published by our research group [20]. The control diet contained soybean meal, sorghum, soybean hulls, dextrin, soy oil, vitamins and minerals. The HSF diet was composed of soybean meal, sorghum, soybean hulls, dextrin, sucrose, fructose, lard, vitamins and minerals plus 25% sucrose in drinking water. Nutritional composition of both diets is presented in Table 1, without considering drinking water.

### 2.2. Nutricional Profile

Feed consumption (FC) was measured daily and body weight (BW) was assessed weekly. Caloric intake (CI) for the control group was calculated according to the following formula: caloric intake (kcal/day) = feed consumption (g) × dietary energy (3.59 kcal/g). For the animals that received the HFS diet, the energy intake was calculated according to the formula: volume consumed (mL) × 0.25 (equivalent to 25% fructose) × 4 (calories per gram of carbohydrate) + caloric values offered by feeding (feed consumption (g) × dietary energy (4.35 kcal/g). Feed efficiency (FE) is the ability to convert caloric intake to BW and it was determined as follows: FE(%) = BW gain (g)/total caloric intake (kcal) × 100 [16].

### 2.3. Plasma Measurements

After 12-h fasting, blood was collected from the tail and the plasma was used for biochemical analysis. Plasma glucose was determined by using a glucometer (Accu-Chek Performa; Roche Diagnostics, Indianapolis, IN, USA). Insulin level was measured by enzyme-linked immunosorbent assay (ELISA) method using commercial kits (Millipore). Triglycerides, total cholesterol, high-density lipoprotein (HDL), urea, and creatinine were measured by an automatic enzymatic analyzer system (Biochemical analyzer BS-200, Mindray, China). Non-HDL cholesterol fraction (VLDL + IDL + LDL) which is considered an estimation of the total atherogenic particles in plasma, was calculated by the formula: (non-HDL Chol = total Cholesterol-HDL) [21].

### 2.4. Homeostatic Model Assessment Index (HOMA-IR)

The homeostatic model of insulin resistance (HOMA-IR) was used as an insulin resistance index, and it was calculated using the following formula: HOMA-IR = [fasting glucose (mmol/L) × fasting insulin (µU/mL)]/22.5 [16].

### 2.5. Systolic Blood Pressure (SBP)

SBP evaluation was assessed in conscious rats by the non-invasive tail-cuff method with a NarcoBioSystems^®^ Electro-Sphygmomanometer (International Biomedical, Austin, TX, USA). The animals were warmed in a wooden box (50 × 40 cm) between 38–40 °C with heat generated by two incandescent lamps for 4–5 min to cause arterial vasodilation in the tail and were then transferred to an iron cylindrical support that was specially designed to allow total exposure of the animal’s tail [22]. After this procedure, a cuff with a pneumatic pulse sensor was attached to the tail of each animal. The cuff was inflated to 200 mmHg pressure and subsequently deflated. The blood pressure values were recorded on a Gould RS 3200 polygraph (Gould Instrumental Valley View, OH, USA). The average of two pressure readings was recorded for each animal.

### 2.6. Echocardiographic Analysis

At 20th week, all the animals were evaluated in vivo by transthoracic echocardiography, using a Vivid S6 system equipped with multifrequency ultrasonic transducer 5.0 to 11.5 MHz (General Electric Medical Systems, Tirat Carmel, Israel). All exams were performed by the same examiner and obtained according to the leading-edge method recommended by the American Society of Echocardiography. Rats were lightly anesthetized by intramuscular injection with a mixture of ketamine (50 mg/kg) and xylazine (1 mg/kg). After shaving their chest, the animals were placed in left decubitus position. To implement structural measures of the heart, the images were obtained in one-dimensional mode (M-mode) guided by the images in two-dimensional mode with the transducer in the parasternal position, minor axis. Left ventricular (LV) evaluation was performed by positioning the cursor M-mode just below the mitral valve plane at the level of the papillary muscles. The images of the aorta and left atrium were obtained by positioning the M-mode course to plan the level of the aortic valve. The following cardiac structures were measured: diastolic diameter (LVDD) and systolic (LVSD) LV; diastolic thickness posterior wall of the left ventricle (LVPWD); diameter of the aorta (DA) and left atrium (LA). The relative thickness of the LV (ERVE) was calculated by dividing LVPWD multiplied by two by LVDD. Left ventricular mass (LVM) was calculated using the formula [(LVDD +LVPWD IVSDT) 3 − (LVDD) 3] × 1.04 where 1.04 is the specific density of the myocardium. MVE index (LVMI) was calculated by normalizing to body weight Estimated LV mass. The LV systolic function was assessed by the following parameters: percentage of endocardial shortening (Δ% endo) [(LVDD − LVSD)/LVDD] × 100; midwall fractional shortening (% Δ meso) {[(LVDD + ½ + ½ LVPWD IVSDT) − (LVSD + ½ + ½ IVSST LVPWS)]/(LVDD + ½ + ½ LVPWD IVSDT)} × 100; shortening velocity rear wall (LVPW), which is the maximum tangent of the stroke movement of the rear wall. The LV diastolic function was evaluated by the following indices: peak velocity of early diastolic filling (E wave); peak velocity of late diastolic filling (A wave); ratio between the E and A waves (E/A); deceleration time of E wave (DTE); isovolumetric relaxation time in absolute values (IRT) and normalized for heart rate (TRIVn = IRT/R-R0,5). The joint assessment of diastolic and systolic LV function was performed by myocardial performance index also known as Tei index (sum of isovolumetric contraction and IRT time, divided by the left ventricular ejection time). The study was supplemented by evaluation by tissue Doppler systolic displacement (S’), early diastolic (E’), and late (A’) of the mitral annulus (arithmetic average travel speeds of lateral and septal walls), and the ratio by the waves E and E’(E/E’) [23,24].

### 2.7. Statistical Analysis

Data are presented as mean ± standard deviation (SD) or median (interquartile range). Differences between the groups were determined by using Students-t test for independent samples. Pearson correlation was used for analytical statistic for association between cardiac parameters and metabolic variables. All statistical analyses were performed using SigmaStat for Windows (Version 3.5). *p* value < 0.05 was considered as statistically significant.

### 2.8. Results

**Figure 1:**
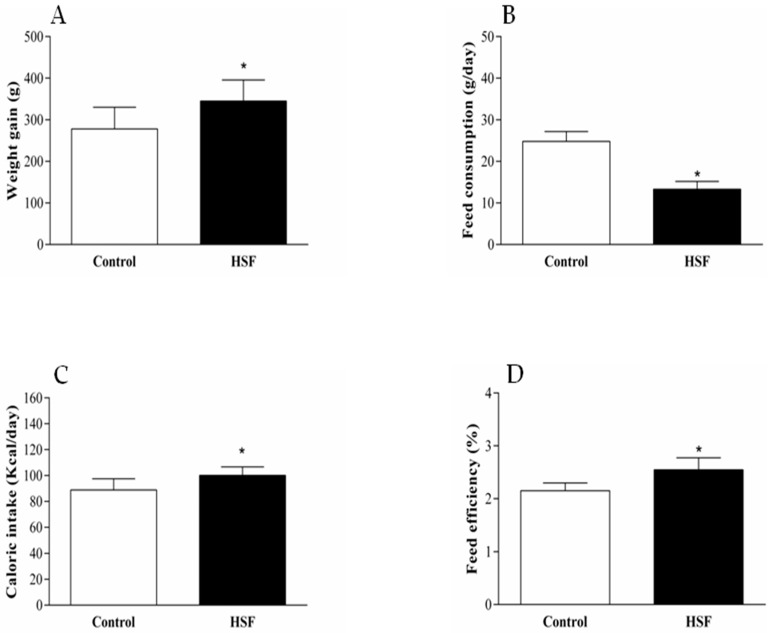
Nutritional profile of the groups. (**A**) weight gain (g); (**B**) feed consumption (g/day); (**C**) caloric intake(kcal/day); (**D**) feed efficiency (%). HSF—high-sugar high-fat group. * indicates *p* < 0.05.

After 20 weeks of treatment simulating a western diet, the HSF group showed higher weight gain than the control group. Even with a lower feed consumption, the HSF group showed higher caloric intake that resulted in higher feed efficiency (Figure 1). 

In addition, the western diet induced changes in glucose metabolism homeostasis, characterized by higher glucose, insulin and HOMA-IR compared to the control group (Table 1).

**Figure 2:**
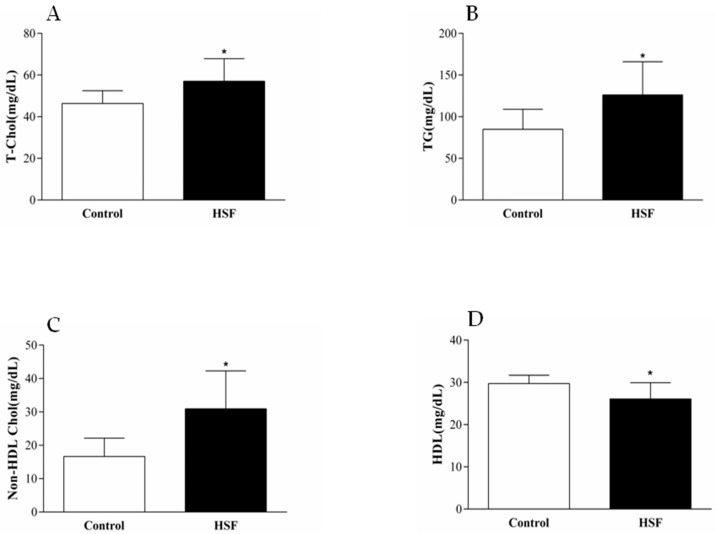
Effect of HSF diet on lipid profile. (**A**) T-Chol: Total Cholesterol (mg/dL); (**B**) TG: Triglycerides (mg/dL); (**C**) Non-HDL Chol:(mg/dL); (**D**) HDL: High-density lipoprotein (mg/dL). Control group; HSF-high-sugar high-fat group. * indicates *p* < 0.05.

The western diet was also associated with systolic and diastolic cardiac dysfunction, and remodeling at 20th week (Table 2), and changes in plasma lipid profile-higher TG, TC, non-HDL, and lower HDL (Figure 2).

The cardiac variables and systolic blood pressure are presented in Table 3. The diet promoted an increase in systolic blood pressure, cardiac remodeling, and deterioration of cardiac function, visualized by echocardiogram analysis.

**Figure 3:**
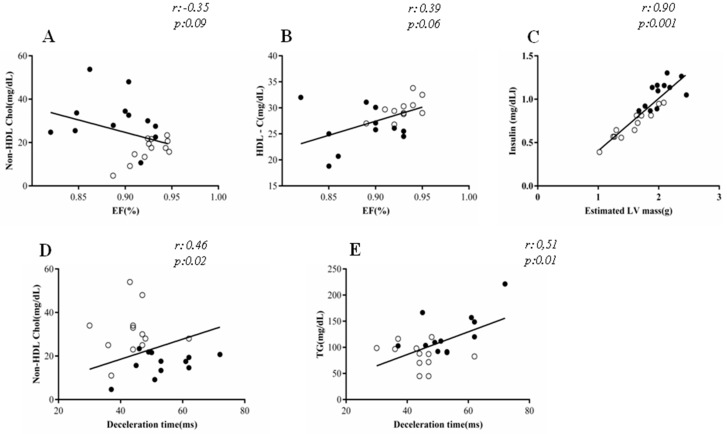
Correlation between echocardiographic and biochemical parameters. EF: Ejection Fraction; (**A)** Non-HDL Chol:(mg/dL)/EF; (**B**) TG: HDL: High-density lipoprotein(mg/dL)/EF; (**C**) Insulin:(mg/dL)/estimated LV mass; (**D**) Non-HDL Chol:(mg/dL)/Decelaration time and (**E**) Triglycerides (mg/dL)/Decelaration time. Control group (○), HSF group (●); Pearson regression was used to examine the associations between variables.

Moreover, these cardiac changes were related to the altered lipid profile (Figure 3). Ejection fraction was inversely correlated with non-HDL cholesterol (*r* = 0.35; *p* = 0.09) and directly correlated with HDL cholesterol (*r* = 0.39, *p* = 0.06), although marginally significant. Positive correlation was also found among the following parameters: insulin vs. estimated LV mass (*r* = 0.90, *p* = 0.001); non-HDL cholesterol vs. deceleration time (*r* = 0.46, *p* = 0.02); TG vs. deceleration time (*r* = 0.50, *p* = 0.01). 

## 3. Discussion

There is a lack of studies in the literature regarding cardiac function injuries and remodeling in obesity/MS models induced by western diets. The present study showed that the western diet model used was able to induce obesity, MS, and cardiac dysfunction and remodeling in the HSF group, even with a lower feed intake than that observed in control group. Since western diet combines high levels of fat and sugar, resulting in tasty but caloric food, the results can be explained by the better feed efficiency in the HSF group. This diet is the closest equivalent to the human ultra-processed food diet (western diet), and provided the animals with varied nutrients, high energy, and palatability, thereby mirroring the key obesogenic features of the human diet [25].

The development of obesity, characterized in this study by the significant difference in body weight, has occurred in the sixth week of experimental treatment and remained for more 14 weeks, with the HSF group presenting higher values compared to the control group. This result shows that the western diet used was efficient to promote obesity in the experimental period of 20 weeks. According to the literature, western diet/ hypercaloric diets are associated with higher body weight in rodents [12,18,26,27]. Besides, the HSF animals in this study also presented many disorders similar to human obesity-related comorbidities, such as hypertension, dyslipidemia hypertriglyceridemia, glucose intolerance, insulin resistance, and hyperinsulinemia. Diets with high sugar and fat are extensively used to induce obesity and MS [28,29,30], hyperinsulinaemia, hyperglycemia and hepatic steatosis [15], dyslipidemia [31], and elevated blood pressure [32,33,34,35,36].

The cardiac morphological analysis in the current study revealed that the western diet leaded to hypertrophy, as characterized by increased left atrium, aorta diameter, left ventricular diastolic and systolic diameter, estimated mass of left ventricle and relative wall thickness. Moreover, it was also observed cardiac dysfunction, with decreased ejection fraction, shortening Δ% endo, and increased deceleration time. All these results show the robustness of the diet to induced cardiac disorders, since most of the experimental studies relating cardiac function and diet- induced obesity present divergent results. Some studies using echocardiogram analyses did not find cardiac dysfunction in the obesity model [12,13], whereas, other authors only demonstrated mild changes [15,37,38].

It is known that overweight and obesity can directly and indirectly modulate the heart, either by promoting an increased hemodynamic overload and neurohumoral activation, or by the secretion of proinflammatory adipokines [15,22,39,40,41,42]. This initial process of cardiac remodeling may be considered as the first step in the sequence of adaptive responses from heart to the stress leaded by a large number of physiological and pathological conditions, as changes in the volume and pressure loads and/or metabolic changes [43,44,45]. Rider et al. proposed that cardiac remodeling is an adaptive characteristic of obesity [46]. Thus, obesity-induced changes in cardiac structure may be elicited directly by obesity-induced increases in cardiac loading conditions (preload and afterload) or indirectly by obesity-induced cardiometabolic abnormalities such as dyslipidaemia and insulin resistance/diabetes [47,48]. The literature reports that the insulin resistance induced by obesity with associated hyperinsulinemia could promote cardiac remodeling via the growth-promoting properties of insulin or by attenuating the anti-apoptotic signaling of the phosphatidylinositol 3′-kinase (PI3K)-Akt (protein kinase B [PKB]) pathway elicited by insulin receptor activation [46,48]. Considering the strong correlation between insulin and left ventricular estimated mass found in this study, probably this pathway was activated in the animals. Clinical studies involving diabetes type 2, congestive heart failure, and obesity had correlated echocardiographic findings with insulin and lipid profile [49,50,51,52]. However, our model of diet-induced obesity showed an obesity-associated cardiomiopathy and brings new insights related to this condition and metabolic changes sought to elucidate whether this condition is correlated with metabolic changes. 

There are recommendations to maintenance of target values of LDL cholesterol in order to protect against LV remodeling [53]. High-density lipoprotein (HDL) is one of the major lipoproteins in the blood. Studies have demonstrated that plasma HDL levels are inversely correlated with the incidence of coronary heart disease [54]. Post-infarct ejection fraction is lower in patients with low HDL cholesterol levels [55,56,57]. In addition, HDL cholesterol anti-inflammatory properties have been extensively discussed [58]. In vitro and in vivo studies suggest that the expression of Apo A1, the primary protein component of HDL cholesterol, is related with lower expressions of cell adhesion molecules ICAM-1 and VCAM-1 [59,60] and lower expression of NF-kB and TNF-α. Inflammatory cell infiltration and heart tissue inflammation have been implicated on pathophysiology of DCVs [61]. Although HDL cholesterol anti-inflammatory properties on cardiac remain under discussion [62], this is a possible mechanistic pathway for the explanation of the relationship between higher HDL cholesterol levels and an efficient systolic function.

## 4. Conclusions

In summary, this paper brings important findings, in a Wistar rats experimental model, following the consumption of a western diet that promoted cardiac remodeling. The diet employed in the current study was able to induce cardiac disorders related to metabolic parameters. However, more studies to investigate the causal mechanisms are necessary, which will allow the development of a new therapy target for clinical practice.

## Figures and Tables

**Table 1:** Nutritional composition of the diets.

	Diet	
Ingredients	Control	HSF
Soybean meal (g/kg)	335	340
Sorghum (g/kg)	278	80
Soy hulls (g/kg)	188.5	116.7
Dextrin (g/kg)	146.5	20
Sucrose (g/kg)	-	80
Fructose (g/kg)	-	180
Soy oil (g/kg)	14	-
Lard (g/kg)	-	154.3
Minerals (g/kg)	25	25
Salt (g/kg)	4	8
**Components**		
Protein (%)	20	16
Carbohydrate (%)	60	70
Fat (%)	4	14.6
% Energy from protein	22.85	13.45
% Energy from carbohydrate	66.78	58.69
% Energy from fat	10.37	27.8
Energy (kcal/g)	3.59	4.35

HSF diet had 25% of sucrose in drinking water.

**Table 2:** Effect of high-sugar and high-fat (HSF) diet on plasma metabolic parameters.

	Groups	
Variables	Control (*n* = 12)	HSF (*n* = 12)
Glucose (mg/dL)	83.4 ± 6.3	97.9 ± 8.5 *
Insulin (mg/dL)	2.5 ± 1.2	5.2 ± 1.3 *
HOMA-IR	21.3 ± 9.6	50.7 ± 11.2 *

Data presented as means ± SD. Control and high-sugar high-fat (HSF) groups; *n*: animals numbers; HOMA-IR: homeostatic model assessment index; * *p* < 0.05 versus C; Student’s *t*-test for independent samples.

**Table 3:** Effect of HSF diet on hemodynamic and cardiac remodeling.

Variables	Groups	
	Control (*n* = 12)	HSF (*n* = 12)
LVDD, mm	7.50 ± 0.40	6.53 ± 0.49 *
LVSD, mm	2.68 ± 0.34	3.31 ± 0.44 *
LVPWD, mm	1.54 ± 0.11	1.97 ± 0.11 *
Aorta diameter, mm	3.79 ± 0.24	4.01 ± 0.19 *
Left Atrium	4.73 ± 0.20	6.17 ± 0.41 *
Estimated LV mass, g	1.56 ± 0.32	2.03 ± 0.23 *
Relative wall thickness	0.45 ± 0.03	0.58 ± 0.06 *
Systolic volume, mL	23.5 ± 2.8	26.6 ± 5.8
Shortening Δ% endo	58.2 ± 3.3	52.5 ± 55.3 *
Shortening Δ% meso	25.6 ± 2.1	25.3 ± 2.7
Ejection fraction, %	0.92 ± 0.01	0.89 ± 0.03 *
Deceleration time, MS	44.1 ± 7.8	53.4 ± 9.4 *
Ew, m/s	78.9 ± 8.4	77.9 ± 6.6
Aw, m/s	48.7 ± 11.6	45.9 ± 14.1
E/A, m/s	1.67 ± 0.27	1.85 ± 0.64
IRT	22.9 ± 3.1	28.1 ± 4.8 *
Systolic blood pressure, mmHg	126 ± 5	136 ± 5 *

Data presented as means ± SD. Control and high-sugar high-fat (HSF) groups; n: animals numbers; LV:Left ventricular; LVDD:Left ventricular diastolic diameter; LVSD:Left ventricular systolic diameter; LVPWD:diastolic posterior wall thickness; Aw: A-wave mitral inflow velocity; Ew: E-wave mitral inflow deceleration time; IRT: Isovolumetric relaxion time; * *p* < 0.05 versus Control; Student’s *t*-test for independent samples.
